# Is the post-COVID-19 syndrome a severe impairment of acetylcholine-orchestrated neuromodulation that responds to nicotine administration?

**DOI:** 10.1186/s42234-023-00104-7

**Published:** 2023-01-18

**Authors:** Marco Leitzke

**Affiliations:** Department of Anesthesiology, Helios Clinics, Colditzer Straße 48, 04703 Leisnig, Germany

**Keywords:** Post COVID 19 syndrome, Cholinergic neuromodulation, Nicotine, Nicotinic acetylcholine receptors, Vagus nerve signaling

## Abstract

Following a SARS-CoV-2 infection, many individuals suffer from post-COVID-19 syndrome. It makes them unable to proceed with common everyday activities due to weakness, memory lapses, pain, dyspnea and other unspecific physical complaints. Several investigators could demonstrate that the SARS-CoV-2 related spike glycoprotein (SGP) attaches not only to ACE-2 receptors but also shows DNA sections highly affine to nicotinic acetylcholine receptors (nAChRs). The nAChR is the principal structure of cholinergic neuromodulation and is responsible for coordinated neuronal network interaction. Non-intrinsic viral nAChR attachment compromises integrative interneuronal communication substantially. This explains the cognitive, neuromuscular and mood impairment, as well as the vegetative symptoms, characterizing post-COVID-19 syndrome. The agonist ligand nicotine shows an up to 30-fold higher affinity to nACHRs than acetylcholine (ACh). We therefore hypothesize that this molecule could displace the virus from nAChR attachment and pave the way for unimpaired cholinergic signal transmission. Treating several individuals suffering from post-COVID-19 syndrome with a nicotine patch application, we witnessed improvements ranging from immediate and substantial to complete remission in a matter of days.

**Supplementary Information:**

The online version contains supplementary material available at 10.1186/s42234-023-00104-7.

## Introduction

### Post-COVID-19-syndrome

The coronavirus SARS-CoV-2 evoked pandemic calamity and took a toll on the world’s population, with a death toll of 6 million victims within 30 months (COVID-19 Excess Mortality Collaborators 2022). Unprecedented scientific efforts led to a better understanding of the viral structure, transmission pathways and pathologic patterns, which ultimately helped to create sufficiently protective vaccines. The pathogen, however, always seems to be one step ahead; genetic variants of SARS-CoV-2 (Weisblum et al. 2020; Tang et al. 2021; Harvey et al. 2021; Mohiuddin and Kasahara 2022; Vaughan 2021; Karim and Karim 2021) present higher contagiousness (Karim and Karim 2021), compromise the sufficiency of vaccines (Harvey et al. 2021), promote escape from natural immunity (Harvey et al. 2021; Karim and Karim 2021) or reveal new pathology patterns (Abdelnabi et al. 2021).

Meanwhile, we are becoming more and more aware that even after convalescence from acute COVID-19, the suffering in many cases is not yet over (Rimmer and Covid-19, 2020). Symptoms such as chronic fatigue (Baker et al. 2021; Lamprecht 2020; Yelin et al. 2020), dizziness (Baker et al. 2021; Heneka et al. 2020), low-grade fever (Yelin et al. 2020), anosmia (Lee et al. 2020), memory lapses (Yelin et al. 2020), ageusia (Lee et al. 2020), muscle weakness (Yelin et al. 2020), diarrhea and bouts of vomiting (Yelin et al. 2020), concentration and sleep difficulties (Baker et al. 2021; Yelin et al. 2020), mood disorders (Yelin et al. 2020), headache (Baker et al. 2021; Yelin et al. 2020), cognitive impairment (Stam et al. 2020), motor deficits, new onset of diabetes (Yelin et al. 2020; Draulans 2020; Rubino et al. 2020) and hypertension (Yelin et al. 2020), dyspnea (Baker et al. 2021; Yelin et al. 2020; Stam et al. 2020) and exercise intolerance (Heneka et al. 2020; Stam et al. 2020) are summarized as post-COVID-19 syndrome (Lamprecht 2020) (Table 1). The occurrence of the mentioned symptoms weeks or even months after the acute phase of SARS-CoV-2 (Yelin et al. 2020) infection is thereby independent of the severity of the initial disease course (Tenforde et al. 2020; Barker-Davies et al. 2020) or baseline chronic medical conditions (Tenforde et al. 2020; Yong 2021). Its incidence is estimated between 35% (outpatients) (Tenforde et al. 2020) and 87% (inpatients) (Carfì et al. 2020) of all individuals experiencing SARS-CoV-2 infection. In addition, the duration of the symptoms is unpredictable (Yelin et al. 2020; Barker-Davies et al. 2020; Sawadogo et al. 2020); after six months, an average of 14 persistent symptoms is reported by subjects suffering from long-haul COVID (Carod Artal 2021).

**Table 1:** Common symptoms of post-COVID-19 syndrome

Post-COVID-19-syndrome related symptoms	Referenced in
Chronic fatigue	Rimmer and Covid-19, 2020; Baker et al. 2021; Lamprecht 2020; Yelin et al. 2020; Yong 2021; Carfì et al. 2020; Klitzman 2020; Goërtz et al. 2020; Sher 2021; Vink and Vink-Niese 2020; Huang et al. 2021; Mendelson et al. 2020; Ortelli et al. 2021; Mardani 2020)
Dizziness	Baker et al. 2021; Heneka et al. 2020; Goërtz et al. 2020; Sher 2021; Görlinger et al. 2020; Dani et al. 2021)
Dyspnea	Baker et al. 2021; Yelin et al. 2020; Stam et al. 2020; Yong 2021; Carfì et al. 2020; Klitzman 2020; Goërtz et al. 2020; Sher 2021; Vink and Vink-Niese 2020; Mendelson et al. 2020; Dani et al. 2021)
Low-grade fever	Yelin et al. 2020; Goërtz et al. 2020; Mendelson et al. 2020)
Anosmia	Rimmer and Covid-19, 2020; Lee et al. 2020; Yong 2021; Goërtz et al. 2020; Sher 2021)
Ageusia	Rimmer and Covid-19, 2020; Lee et al. 2020; Yong 2021; Goërtz et al. 2020; Sher 2021)
Memory lapses	Yelin et al. 2020; Vink and Vink-Niese 2020; Alonso-Lana et al. 2020)
Muscle pain/weakness	Rimmer and Covid-19, 2020; Baker et al. 2021; Lamprecht 2020; Yelin et al. 2020; Yong 2021; Sawadogo et al. 2020; Goërtz et al. 2020; Vink and Vink-Niese 2020; Huang et al. 2021; Mendelson et al. 2020; Ortelli et al. 2021; Mardani 2020)
Diarrhea	Yelin et al. 2020; Yong 2021; Goërtz et al. 2020; Dani et al. 2021)
Vomiting	Yelin et al. 2020; Goërtz et al. 2020; Vink and Vink-Niese 2020)
Concentration difficulties	Rimmer and Covid-19, 2020; Lamprecht 2020; Yelin et al. 2020; Goërtz et al. 2020)
Sleep difficulties	Baker et al. 2021; Yelin et al. 2020; Goërtz et al. 2020; Huang et al. 2021; Mardani 2020; Alonso-Lana et al. 2020)
Mood disorders	Yelin et al. 2020; Yong 2021; Sher 2021; Vink and Vink-Niese 2020; Huang et al. 2021; Mendelson et al. 2020; Ortelli et al. 2021; Mardani 2020; Dani et al. 2021)
Headache	Baker et al. 2021; Heneka et al. 2020; Yong 2021; Sawadogo et al. 2020; Goërtz et al. 2020; Sher 2021; Mendelson et al. 2020; Liu et al. 2020)
Chest tightness/pain	Lamprecht 2020; Yong 2021; Goërtz et al. 2020; Vink and Vink-Niese 2020; Mendelson et al. 2020; Dani et al. 2021; Staats et al. 2020)
Heart palpitations	Baker et al. 2021; Lamprecht 2020; Yelin et al. 2020; Goërtz et al. 2020; Vink and Vink-Niese 2020; Dani et al. 2021; Puntmann et al. 2020)
Cognitive impairment	Baker et al. 2021; Heneka et al. 2020; Yong 2021; Klitzman 2020; Goërtz et al. 2020; Sher 2021; Mendelson et al. 2020; Novak 2020)
Motor deficits	Heneka et al. 2020; Mendelson et al. 2020; Ortelli et al. 2021; Rábano-Suárez et al. 2020)
Exercise intolerance	Yelin et al. 2020; Heneka et al. 2020; Stam et al. 2020; Vink and Vink-Niese 2020; Huang et al. 2021)
New onset of diabetes	Yelin et al. 2020; Draulans 2020; Rubino et al. 2020)
New onset of hypertension	Lamprecht 2020)

These facts underline the enormous significance of post-COVID-19 syndrome to global societies in terms of public health, as well as the political, sociopolitical and financial burdens to respective systems (Stam et al. 2020; Klitzman 2020; Farsalinos et al. 2020; Scordo et al. 2021); the individual somatic and psychological misery of each suffering patient must not be forgotten*.* Thus, we must be aware of this inevitable aftershock to health care systems (Rimmer 2020; Stam et al. 2020) which is to be expected from this chronic form of COVID-19 (Higgins et al. 2021; Phillips and Williams 2021). We will see many more infected patients recovering from the acute phase of COVID-19 along with a large population needing therapy and rehab capacity (Stam et al. 2020; Barker-Davies et al. 2020; Klitzman 2020) to cure the symptoms of the chronic phase (Barker-Davies et al. 2020), better known as post-COVID-19-syndrome (Nath 2020).

### Is it just the ACE2 receptor?

For the acute infection phase, physicians are lacking a causal therapeutic strategy to challenge the viral assault on human organ systems and must confine themselves to symptomatic therapeutic approaches for their patients. In severe cases of SARS-CoV-2 infections, these options prove rather underwhelming (Iyer et al. 2020; Jeong et al. 2020), while the therapeutic situation remains vague regarding post-COVID-19 syndrome (Rimmer, 2020; Carod Artal 2021). The cause of its multifaceted symptomatology is widely speculated, with ongoing systemic inflammation (Heneka et al. 2020; Carod Artal 2021), peripheral organ dysfunction (Heneka et al. 2020) as well as cerebrovascular changes (Baker et al. 2021), viral encephalitis (Heneka et al. 2020) and myalgic encephalomyelitis/chronic fatigue syndrome (ME/CSF) (Wong and Weitzer 2021)), persistent brainstem dysfunction (Yong 2021) and even psychosomatic disorders (Mardani 2020) playing a potential role. This makes therapeutic approaches to long-haul COVID speculative (Crook et al. 2021; Tirelli et al. 2021) at best with rather dissatisfying effectiveness (Scordo et al. 2021).

Our group recently described the crucial relevance of autonomic balance for the severity of COVID-19 disease courses (Leitzke et al. 2020; Leitzke and Schönknecht 2021) and highlighted the significance of nicotinic acetylcholine receptors (nAChRs) for the limiting regulation of cytokine liberation and virus replication on the transcriptional level, restricting NF-KB action along the cholinergic anti-inflammatory pathway (CAP) (Leitzke et al. 2020; Leitzke and Schönknecht 2021). Profound similarities between highly nAChR affine toxins (i.e., from snakes of the *Ophiophagus* (cobra) and *Bungarus* genera, the G-ectodomains of three *Rabies lyssavirus* (formerly Rabies virus) (RABV) strains (Changeux et al. 2020) or muscarinic toxin-like protein and Cobratoxin (naja siamensis) (Farsalinos et al. 2020)) and SARS-CoV-2 specific proteins (Farsalinos et al. 2020; Changeux et al. 2020) were found by analyzing the toxin’s amino-acid (aa) sequence alignment and comparing it to the motifs in spike glycoprotein (SGP) from SARS-CoV-2 (Farsalinos et al. 2020; Changeux et al. 2020).

Changeux et al. (2020) recently proposed a ´nicotine hypothesis´, which implicates the propensity of SARS-CoV-2 to not only bind to ACE2-receptors (ACE2R) but to nicotinic AChRs, as well (Changeux et al. 2020). Virus particles competing with acetylcholine for nAChR binding in order to enter the human body may lead to primary neuro infection (Changeux et al. 2020; Steardo et al. 2020). Furthermore, among the severe and fatal cases of COVID-19, the proportion of nicotine consumers was significantly lower than non-consumers of nicotine (Miyara, et al. 2020). Since nicotine may protect nAChRs from viral attachment, therapeutic nicotine application was proposed in the management of acute COVID-19 infections (Changeux et al. 2020). This argument is convincingly supported by the cohort study of Hippisley-Cox et al. (2020), with a total of 8.28 million participants (including 19,486 confirmed COVID-19 cases), showing lower odds for COVID-19 infection and COVID-19-related ICU stay in association with smoking (Hippisley-Cox et al. 2020).

Farsalinos et al. (2020) examined and identified a “toxin-like” aa sequence in the receptor binding domain of the SARS-CoV-2 spike glycoprotein (SGP) (*aa 375–390*) which shows significant sequence homology with the neurotoxin homolog NL1, one of the many snake venom toxins interacting with nAChRs (Farsalinos, et al. 2020). Additionally, they performed computational molecular modeling and docking experiments under the usage of 3D structures of the SARS-CoV-2 SGP and the extracellular domain of the nAChR α9 subunit (Farsalinos, et al. 2020). Thus, they could show the main interaction between the *aa 381–386* sequence of the SARS-CoV-2 SGP and the *aa 189–192* sequence of the extracellular domain of the nAChR α9 subunit (Farsalinos, et al. 2020), the core of the “toxin-binding site” of nAChRs (Farsalinos, et al. 2020). Likewise, a similar interaction could be demonstrated between the ligand binding domain of the pentameric α7 nicotinic acetylcholine receptor (α7nAChR) chimera and the SARS-CoV-2 SGP (Farsalinos, et al. 2020). The authors concluded that their findings strongly support the hypothesis of a dysregulation in the nicotinic cholinergic system being a considerable part of COVID-19’s pathophysiology (Farsalinos, et al. 2020).

### The pivotal neuromodulation role of nicotinic acetylcholine receptors

Within the central nervous system (CNS), acetylcholine (ACh) is released mainly from projection neurons (PN), which innervate distal areas, and local interneurons interspersing their cellular targets. PNs are found in several nuclei, including the medial habenula, pedunculopontine and laterodorsal tegmental areas, as well as the basal forebrain complex and the medial septum (Reviewed in (Picciotto et al. 2012)). They promote wide and diffuse innervation of numerous neurons in the CNS, and their signaling is carried out by ACh coupling to pre- and post-synaptic, as well as axonal and cell-body located, AChRs on a huge number of targeted neurons throughout the brain (Reviewed in (Picciotto et al. 2012)). Regulating the velocity and amount of transmitter release into the synaptic cleft, they improve the signal-to-noise-ratio (Reviewed in (Picciotto et al. 2012)) and orchestrate fine-tuned, synchronized response behavior of central and autonomic nuclear regions of the brain to internal and external stimuli (Reviewed in (Picciotto et al. 2012)). Moreover, they are involved in synaptic plasticity, neuronal development and learning processes in general (Reviewed in (Picciotto et al. 2012)).

AChRs are categorized into either metabotropic muscarinic (mAChRs) (Wess 2003; Jones, et al. 1992) or ionotropic nicotinic acetylcholine receptors (nAChRs) (Gotti et al. 2006; Hurst et al. 2013). In addition to their different propensity in binding to either muscarine or nicotine (Jones, et al. 1992), they differ in their signaling properties; great differences are observed in the signal transmission velocity (Jones, et al. 1992). Signal transduction of mAChRs is realized slowly via coupling to G-proteins—either activating phospholipase C (PLC) or inhibiting adenylate cyclase (Scarr 2012)—or non-canonically (Scarr 2012), altering pathways involving phospholipase A2, phospholipase D and tyrosine kinase, in addition to calcium channels (Scarr 2012). Either excitatory or inhibitory behavior of the mAChR effect is dependent on the targeted cell type to which muscarinic cholinergic signaling is applied (Scarr 2012). This diversity of mAChRs in terms of their several modes of action, together with the high degree of homology at the orthosteric ACh-binding site (Scarr 2012), made the development of specifically-acting ligands which could therapeutically influence muscarinic AChR-related signaling pathways almost impossible until recently (Wess 2003; Scarr 2012).

**Fig. 1:**
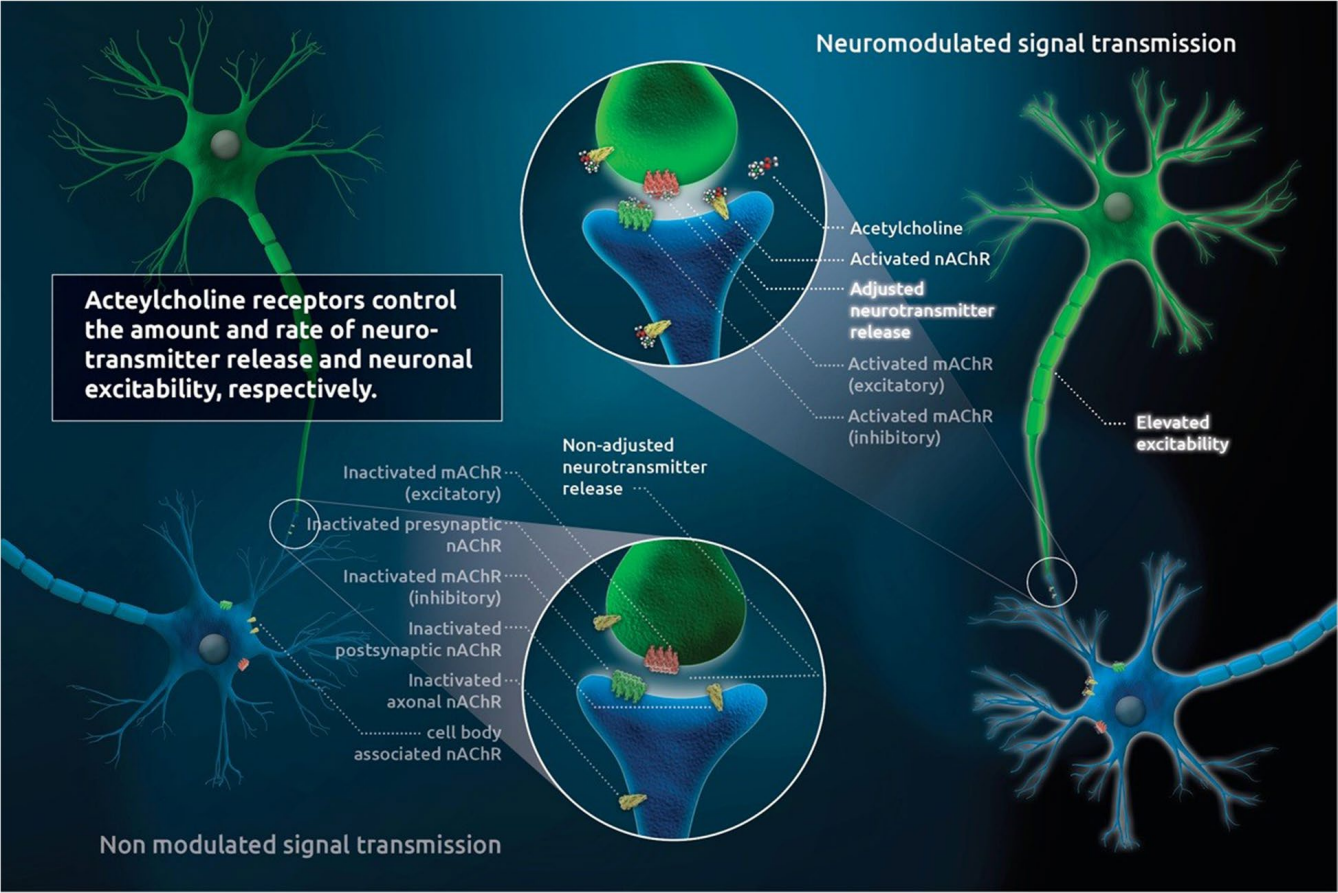
(Cholinergic neuromodulation): AChRs are located in the membranes of neural cell bodies, as well as in pre- or post-synaptic sites and at the axonal membranes. ACh binding regulates both the velocity and amount of transmitter release, as well as cell excitability; it also orchestrates network operation between several core groups, as well as synchronized response behavior to internal and external stimulation. Cholinergic neuro-modulatory action is an indispensable requirement for neural plasticity, neuronal development and learning processes. Thus, numerous physiological functions (sleep, arousal, fatigue, anxiety, nutritional behavior, cognition and central processing of pain) are interactively regulated by cholinergic neuromodulation. The two different subtypes of AChRs bind, despite ACh, to either nicotine (nAChrs) or muscarine (mAChRs). While mAChRs act slowly and promote a multitude of excitatory or inhibitory transmission effects via numerous canonical and non-canonical pathways, nAChRs consist of a homomeric (α) or heteromeric (α/β) configuration of 5 subunits, forming calcium channels with fast reaction to agonistic stimulation. These allosteric nAChRs are the principal structures of central and autonomic neuromodulation and underlie great plasticity in terms of count, binding sites and affinity dependent upon agonist stimulation

In contrast, nAChR activation leads to fast and non-selective opening of membrane-bound, excitatory cation channels (Jones, et al. 1992). These pentameric nAChRs (Gotti et al. 2006) with allosteric configuration (Spurny et al. 2015) are an equally essential part of the interneuronal communication within the CNS and the autonomic nervous system (ANS) (Picciotto et al. 2012). Even though neuromodulators commonly act in a metabotropic fashion, ionotropic nAChRs have been shown to act largely neuro-modulatory, as well (Picciotto 2003). They consist of a varying, either homomeric or heteromeric, combination out of nine (α2-α10) α- and/or three (β2-β4) β-subunits (Gotti et al. 2006; Gotti and Clementi 2004; Lloyd and Williams 2000) and are located at presynaptic or pre-terminal membrane sections where they modulate transmitter release. In addition, nAChRs are found on dendrites or neuronal cell bodies, where they generate postsynaptic effects (Gotti et al. 2006). In the CNS, nAChR neuromodulation realizes the regulation of transmitter release, cell excitability and integrative adaptation of neuronal activity (Gotti et al. 2006) (Fig. 1). Stimulation of nAChRs can increase the release of several neurotransmitters, such as glutamate, gamma-aminobutyric acid (GABA) and dopamine (DA) (Reviewed in (Picciotto et al. 2012)). Thus, networking and coordination of essential physiological functions such as arousal, sleep, fatigue, anxiety, nutritional behavior, cognition and central processing of pain (Gotti et al. 2006; Gotti and Clementi 2004; Hogg et al. 2003; Dajas-Bailador and Wonnacott 2004; Hogg and Bertrand 2004) are regulated. nAChRs therefore play a central role in the synchronization of neuronal activity (Picciotto et al. 2012; Picciotto 2003).

Gotti et al. (2006) described the α4β2 nAChR subtype as the best-characterized nAChR in an animal (rat) brain in their review (Gotti et al. 2006). They stated that this nicotinic AChR is the principal neuro-modulatory nAChR subtype in several cerebral subregions, such as the cortex, striatum, superior colliculus, lateral geniculate nucleus and cerebellum (Gotti et al. 2006). This was demonstrated in the detectable loss of high-affinity nAChRs in the CNS of α4β2 subunit knockout mice (Picciotto et al. 2001) and underlines the central role of nAChRs in the entire neuro-modulatory network.

### Nicotine effect on the nicotinic acetylcholine receptors

The chronic application of nicotine in animal and in vitro models yielded an up-regulation (Buisson and Bertrand 2001) of respective central binding sites, whereas the chronic increase of the natural ligand ACh via application of a cholinesterase inhibitor led to a consecutive decrease of the central density of nAChRs (Schwartz and Kellar 1983). These changes occur very quickly after nicotine exposure, making it clear that cholinergic signaling adapts rapidly to nicotine and that nicotine can effectively improve compromised cholinergic neurotransmission. These effects were particularly seen in α4β2-type receptors with the aforementioned prominent significance to nicotinic cholinergic neuromodulation (Gotti et al. 2006). It is worth noting that nAChR up-regulation is not accompanied by desensitization but rather an increased ratio of high-affinity nAChRs (from 25% at baseline and increased up to 70% under nicotine exposure) compared to low-affinity nAChRs (Buisson and Bertrand 2001). In addition, the opening frequency of the α4β2 cation channels increases up to three times under chronic nicotine exposure (Buisson and Bertrand 2001). Nicotine exposure therefore leads to functional up-regulation of human α4β2 nAChRs (Buisson and Bertrand 2001).

From a clinical perspective, nicotine application leads to functional improvement of vigilance, locomotor activity, cognition, respiratory function, cortical blood flow, EEG activity and pain resilience, as well as gastrointestinal- and cardiovascular regulation in animals (Lloyd and Williams 2000). French et al. (1999) demonstrated a long-lasting (up to 72 h after nicotine exposure) increase of neurotrophic nerve growth factor (NGF) mRNA after nicotine administration to the hippocampus, suggesting long-term neuroprotective effects of nicotine (French et al. 1999). Nicotine works as a ligand with high affinity and profound intrinsic activity on nAChRs (Gotti et al. 2006), improving the responsiveness (Buisson and Bertrand 2001) and activity (Lloyd and Williams 2000) of these core receptors of neuromodulation substantially.

Apart from the prescription of transcutaneous nicotine application as a substitute for weaning smokers, the transcutaneous application of this substance has been investigated in clinical trials evaluating its therapeutic effects on neurologic or gastrointestinal disorders in non-smoking patients; these investigations showed no substantial side effects (Newhouse et al. 2012; Sandborn 1997; Pullan et al. 1994). Using very high dosages of nicotine (up to 107 mg/day), however, led nearly every patient with more than 90 mg/day to present with frequent nausea and vomiting (Villafane et al. 2007). Nonetheless, all individuals in a trial investigating the ameliorative effects of nicotine on Parkinson’s disease (PD) showed improved motor scores under reduced dopaminergic treatment (Villafane et al. 2007). In contrast to the well-known addictive potential linked to the chronic inhalation of nicotine, none of the trials could show a nicotine dependency after the withdrawal of transcutaneous nicotine application at the end of the investigations (Newhouse et al. 2012; Sandborn 1997; Pullan et al. 1994; Villafane et al. 2007).

### The competition of SARS-CoV-2, acetylcholine and nicotine at the nicotinic acetylcholine receptor

**Fig. 2:**
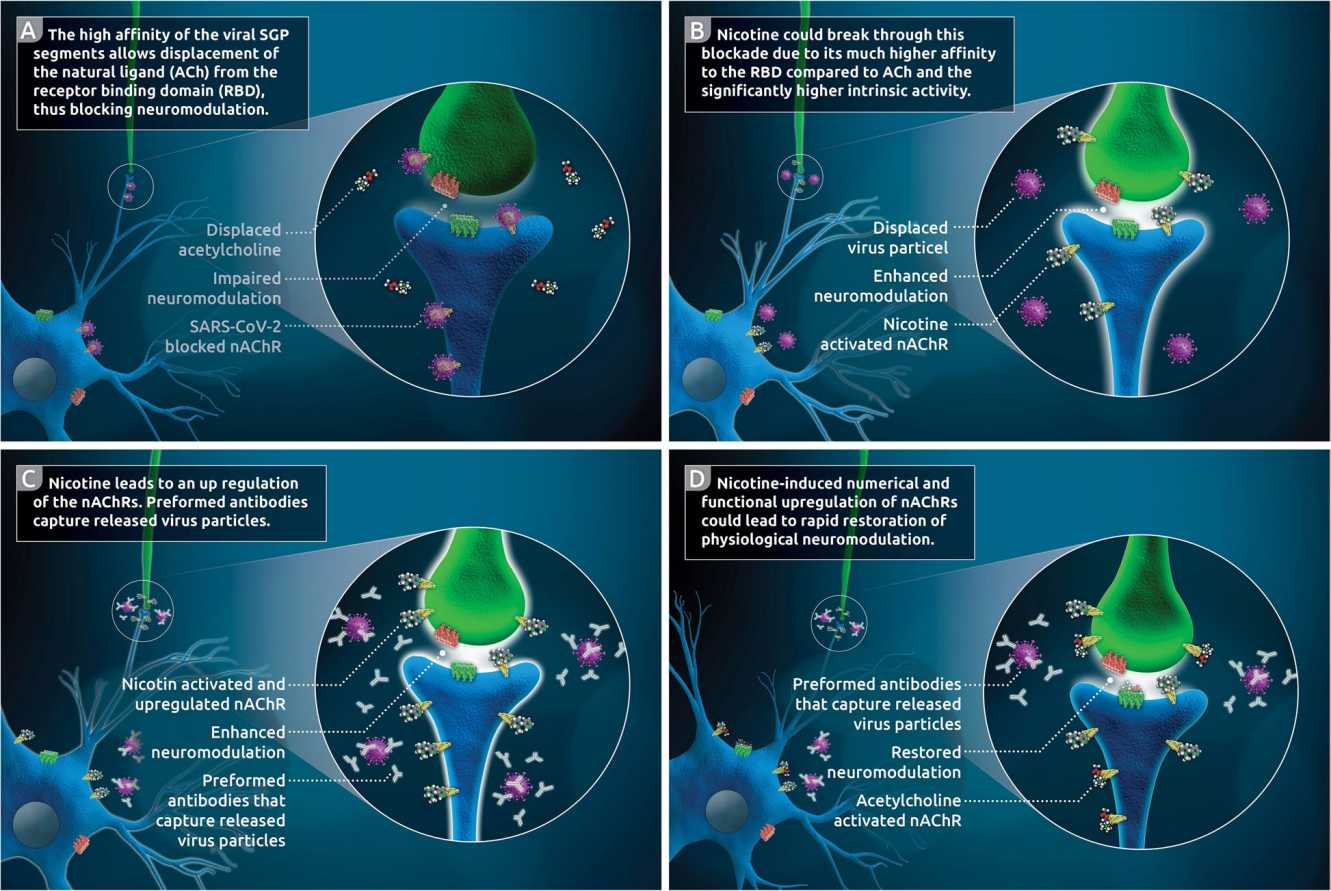
(nAChR competition of ACh, SARS-CoV-2 and nicotine): Membrane-bound neuro-modulatory nAChRs get attached to SARS-CoV-2 viruses in a non-intrinsic fashion, displacing the natural ligand (ACh) and thereby substantially compromising neuro-modulatory cholinergic signaling (**A**). Due to the high affinity of nicotine to nAChRs, the virus is extruded from the attachment to nAChRs by nicotine, thus neutralizing the blockade of cholinergic neuro-modulatory signal transmission (**B**). Since long-haul COVID patients have pre-formed SARS-CoV-2 specific antibodies, the released viruses are captured by these antibodies, thereby preventing active reinfection with SARS-CoV-2 (**C**). Both the high intrinsic activity of nicotine at nAChR and the nicotinic up-regulation of nAChRs lead to the re-establishment of ACh-borne neuromodulation (**D**)

In terms of the central role of nAChRs in interneuronal communication and their involvement in almost every synaptic signal transmission, the possibility that SARS-CoV-2 binds to these nAChRs on a large scale in a non-intrinsic way is a plausible explanation for the widespread symptoms of long-haul COVID-19. By competitively inducing a diminished effect of its natural ligand (ACh), the viral blockade of these receptors leads to a sharp deterioration of cholinergic neuromodulation (Fig. 2A). Thus, most of the long-term COVID-associated deficiencies (Table 1) can be attributed to such neuromodulatory deterioration.

Referring to the above-mentioned results of Changeux et al. (2020), Oliveira et al. (2021) investigated the possible binding of SARS-CoV-2 SGP to nAChRs using molecular simulations of validated, detailed atomic structures of nAChRs and the spike protein (Oliveira et al. 2021). Examining the Y674-R685 loop of the viral SGP and its binding to three different nAChR types (i.e., α4β2, α7 and the muscle-like nAChR αβγδ from Tetronarce californica), their results predict an apparent nAChR affinity of SARS-CoV-2-related spike protein due to a PRRA (proline, arginine, arginine, alanine) motif in the spike binding region. Notably, this is not found in other SARS-like coronaviruses (Oliveira et al. 2021). Using principal component analysis (PCA), molecular mechanics Poisson-Boltzmann surface area (MM-PBSA) approach (Homeyer and Gohlke 2012), and in silico alanine-scanning mutagenesis (Anand et al. 2014), the authors calculated AChR binding related conformational behavior of the receptor protein. Likewise, they calculated subtype-specific different but uniformly stable complex formation between nAChR and SGP (Oliveira et al. 2021). These results confirm the data from Farsalinos et al. (2020), which showed hydrogen bonding and shape-related interaction of the extracellular domain of α9nAChRs and SARS-Cov-2 SGP, as well as SGP coupling to the ligand binding domain of a pentameric α7nAChR chimera using in silico experiments (Farsalinos, et al. 2020).

The affinity of natural or synthetic ligands to several nAChRs varies in dependency on the distinctive nAChR composition from the α- or β-subunits (Gotti et al. 2006). Despite these subtype-specific differences between the agonist ligands, every binding site shows significantly higher inhibition constants (K_i_) for the natural agonist (ACh) compared to nicotine (Reviewed in (Gotti et al. 2006)). In the case of α7-α7 subunit interface, this indicates an up to 30-fold higher affinity (Gotti et al. 1994) of nicotine to respective α7 subunits containing nAChRs compared to the physiological ligand ACh (Gotti et al. 2006).

The far higher affinity of nicotine to the nAChRs in comparison to ACh, coupled with the apparent capability of SARS-CoV-2 to displace ACh from its specific receptors, suggests that nicotine may counteract the viral blockade of nAChRs and displace the virus from the nAChR binding (Fig. 2B,C,D).

## Material

We investigated one female (32 years old) and 3 males (19, 41 and 52 years old, respectively) who suffered from numerous symptoms indicative of post-COVID-19 syndrome following a PCR-confirmed SARS-CoV-2 infection with a subsequent mild course of disease. The patients described weakness, dyspnea, sleep disturbances, dizziness, complete ageusia and anosmia, along with a variety of other symptoms. Except for the youngest, the patients were not able to continue activities of daily life compared to the period before the SARS-CoV-2 infection. As these multiple unspecific symptoms had not improved over a certain time period without signs of acute COVID-19 infection, they visited our outpatient clinic.

## Methods

**Fig. 3:**
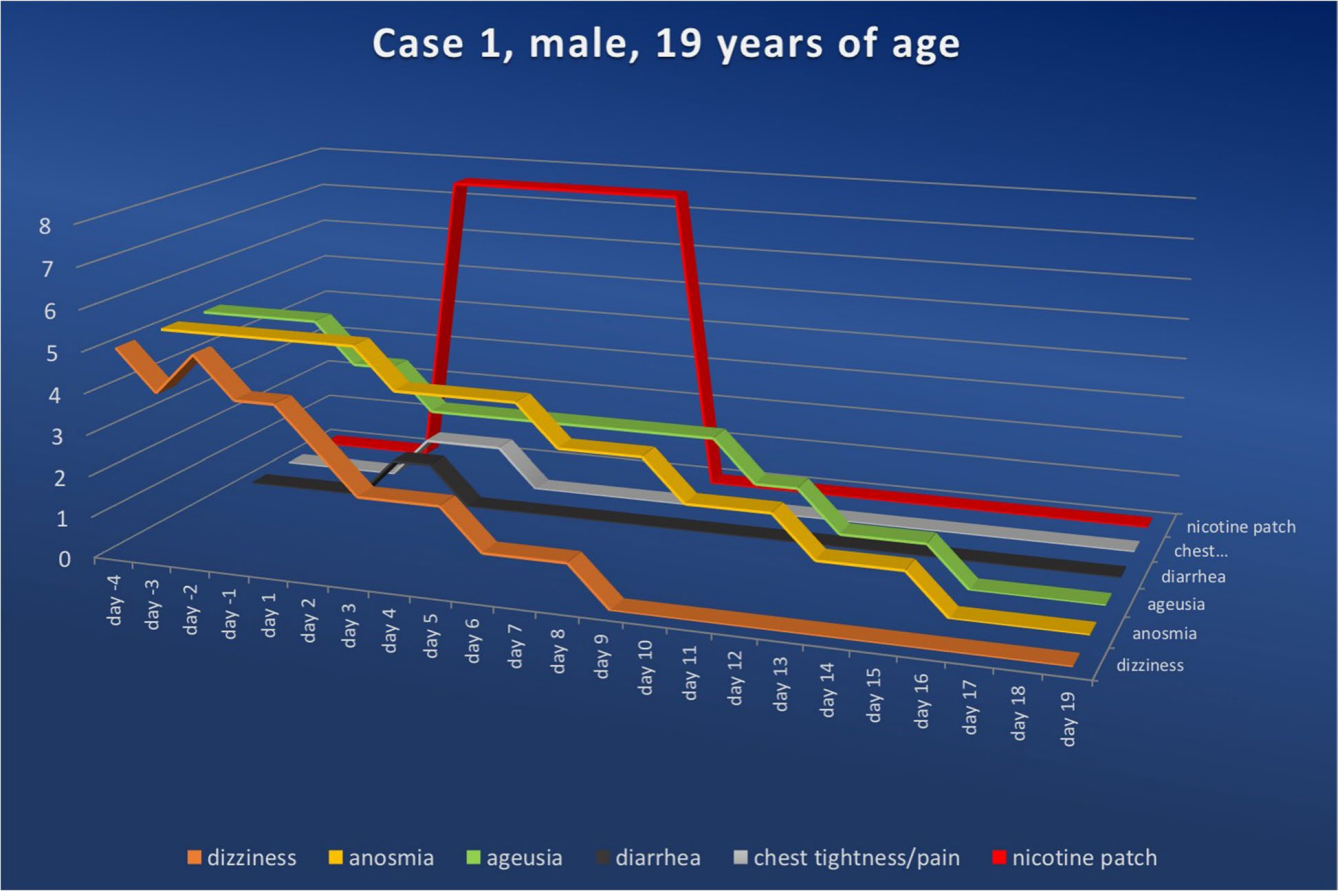
Symptom score for case 1 ranging from the fourth day prior to and to the twenty-sixth day after nicotine administration (fourth symptom-free day). Upon nicotine treatment, the symptom scores in all categories fell continuously. The numerical value in the ordinate shows the value of the symptom score, while the nicotine dosage for the amount delivered per 24 h is shown in red

**Fig. 4:**
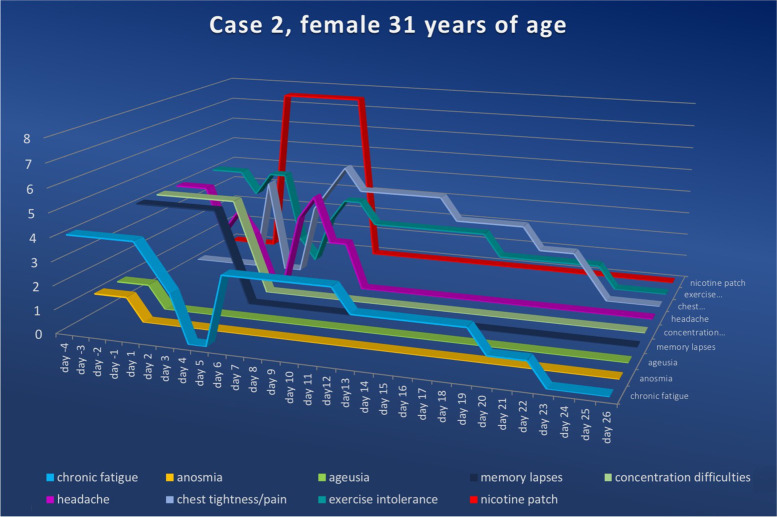
Symptom score for case 2 ranging from the fourth day prior to and to the twenty-sixth day after nicotine administration (fourth symptom-free day). Upon nicotine treatment, the symptom scores in all categories fell continuously. The numerical value in the ordinate shows the value of the symptom score, while the nicotine dosage for the amount delivered per 24 h is shown in red

**Fig. 5:**
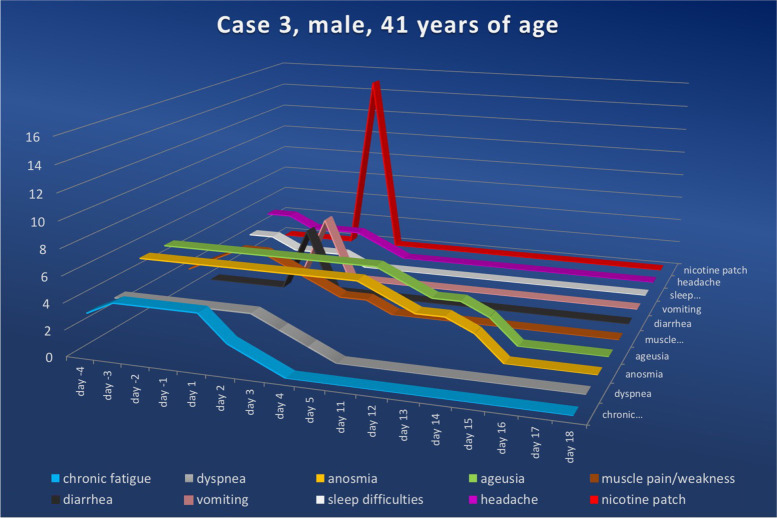
Symptom score for case 3 ranging from the fourth day prior to and to the eighteenth day after nicotine administration (fourth symptom-free day). Upon nicotine treatment, the symptom scores in all categories fell continuously. Despite the mistaken higher dosage and cessation of nicotine application within ten hours of treatment initiation, total remission was achieved on day sixteen. The numerical value in the ordinate shows the value of the symptom score, while the nicotine dosage for the amount delivered per 24 h is shown in red

**Fig. 6:**
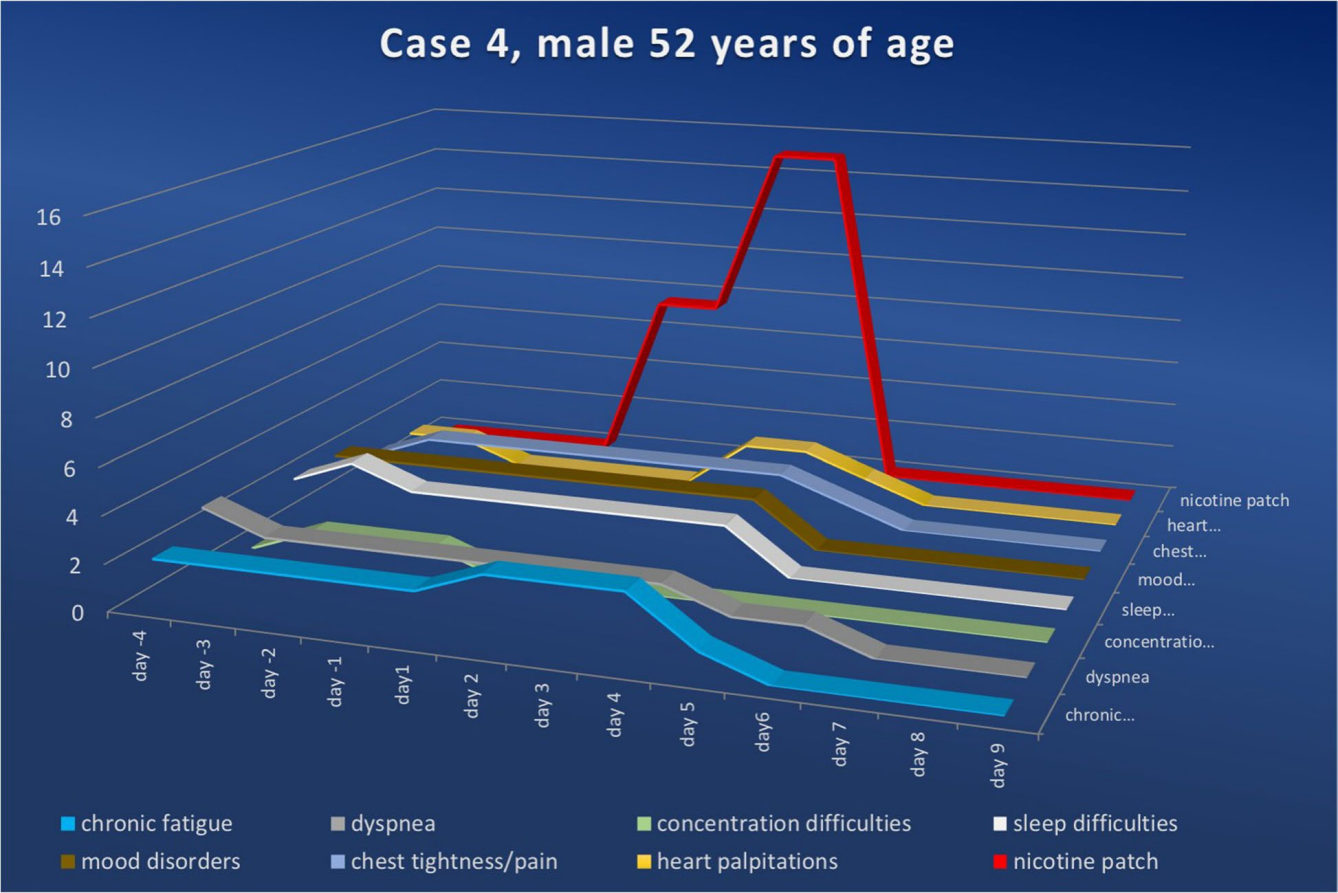
Symptom score for case 4 ranging from the fourth day prior to and to the tenth day after nicotine administration (fourth symptom-free day). Upon nicotine treatment, the symptom scores in all categories fell continuously. The numerical value in the ordinate shows the value of the symptom score, while the nicotine dosage for the amount delivered per 24 h is shown in red

After meticulously explaining the hypothesis described above, as well as the expected effects of nicotine and possible side effects, the patients were advised to apply a standard nicotine patch. Since all included individuals were nicotine-naïve persons, they were instructed to use the lowest available dosage (7.5 mg/24 h) and to administer the patch once daily (in the morning). All patients followed these instructions, except for the 41-year-old patient; he mistakenly purchased the patches in a higher dosage (15 mg/24 h) than recommended. In all cases, we asked patients to register their symptoms starting 4 days before applying the nicotine patch (Figs. 3, 4, 5 and 6) and to score the severity of their complaints on a scale of zero to five daily (Table 2).

**Table 2:** Scoring table to describe the symptom severity of long-haul COVID symptoms

**0**	none
**1**	mild
**2**	clear
**3**	strong
**4**	very strong
**5**	unbearable

## Results

### Case 1

The 19-year-old otherwise healthy patient was diagnosed with SARS-CoV-2 infection by a positive PCR test on March 26^th^, 2021. The patient reported a mild course of the acute infectious disease, with symptoms such as mild fever, sore throat and feelings of weakness, which resolved completely within 10 days. Approximately 3 weeks after the detection of the infection, the patient noticed a sudden loss of his sense of smell and taste, alongside general fatigue. These complaints persisted over the next several months with minimal undulation in symptom severity. On presentation to our outpatient clinic in November of 2021, we counseled the nicotine-naïve patient about the apparent expression of a post-COVID-19 syndrome and informed him about the difficult diagnostic and therapeutic approach to the described symptoms. The patient consented to the off-label use of percutaneous nicotine application and began 24-h applications of nicotine patches (7.5 mg/24 h) for seven days on November 23^rd^, 2021. In the days leading up to nicotine application, weakness was recorded in the range of the two highest possible levels (levels four to five), and anosmia and ageusia were reported at the highest possible level (level five). During nicotine-based treatment, recovery from weakness was most rapid; a daily reduction in symptom severity was achieved, allowing level two to be reached on day three of treatment and maintained for an additional three days. Level one was reported on day six, and by day nine, the patient reported being weakness-free (Fig. 3).

The loss of taste was reduced by one level on the first day of treatment, dropped to level three on the third day, dropped further to level two on the tenth day and reached level one on the thirteenth day. On treatment day sixteen, the patient described the full restoration of his sense of taste.

A similarly protracted symptom reduction was seen with anosmia. Beginning on day three, the patient experienced a reduction from level five to level four, dropping further to level three on day seven, to level two on day ten and reaching level one beginning on day thirteen. From day sixteen, the patient reported being able to smell to the same extent as before his SARS-CoV-2 infection. In a follow-up interview approximately six months post-intervention, the patient reported being symptom-free.

With the onset of nicotine administration, the patient experienced diarrhea for two days, terminating spontaneously and considered mild (level one) by the patient. We interpreted this symptom as a classic side effect of nicotine; it did not require further intervention.

### Case 2

The 31-year-old female patient presented to our outpatient clinic on December 17^th^, 2020, having undergone an acute SARS-CoV-2 infection confirmed by a positive PCR test on November 21^st^, 2020; moderate symptoms included fever, reduction in smell and taste, loss of appetite, headache, pain in the limbs, reduced memory, lack of drive and rhinitis, as well as neck, limb and back pain. The acute infection phase lasted until December 5^th^, 2020 when convalescence was confirmed via a negative PCR test. From that time onwards, she had numerous symptoms, such as chronic fatigue (level four), loss of smell and taste (level one), marked difficulty concentrating (level four), headache (level four) and considerable exercise intolerance (level four). Information, education, informed consent and nicotine therapy were given to the otherwise healthy, nicotine-naïve patient as described above (7.5 mg/24 h) for 6 days.

Starting on day two after the initiation of nicotine therapy, the patient reported a reduction in fatigue of one level per day; fatigue was completely reversed by day four. From the sixth day onwards, however, the patient experienced a recurrence of fatigue to a lesser extent (level three), which then progressed as follows: day thirteen (level two), day twenty (level one) and day twenty-three (level zero). The patient’s reduced retentiveness was perceived as very high (level four) before and at the beginning of treatment; it dropped significantly from the third day following nicotine administration (level two) and was no longer perceptible from the fourth day onward. In the same manner, the ability to concentrate was impaired until the concentration performance perceived before SARS-CoV-2 was regained from the fourth day after the initiation of nicotine therapy.

Similarly, the markedly impaired exercise tolerance (level four) dropped significantly on day three (level one), becoming unreproducible on day four, but rising again slightly on days five (level two) and six (level three), only to fall continuously thereafter from day eight. From the twenty-fourth day after nicotine application, the patient reported full recovery of her physical performance.

Starting on day two, the patient experienced a very unpleasant feeling of tightness in the thoracic region, which was reported to be undiminished (levels three to five) until day thirteen after the start of nicotine therapy; it decreased continuously from then on (level three on day fourteen, level two on day nineteen, level one on day twenty-two) until complete remission was achieved on day twenty-three. We attributed this symptom, which began directly with the start of nicotine administration, to a side effect of the active substance, nicotine. The patient considered these symptoms to be associated with nicotine, as well, which is why she elected to stop the nicotine therapy on day six as opposed to continuing until day seven as we had recommended. This decision was due to the otherwise very good symptom remission until the fourth day of nicotine administration (all symptoms at level zero). In a telephone interview after approximately six months, the patient confirmed that there had been no recurrence of her symptoms.

### Case 3

A 41-year-old male patient visited our outpatient clinic on December 20^th^, 2022, having been ill from a moderate SARS-CoV-2 infection (confirmed on November 13^th^, 2020); his symptoms had been weakness, fever, chills, headache, coughing attacks, loss of sense of smell and taste, shortness of breath, exercise intolerance, permanent fatigue and a pronounced feeling of weakness.

At the time of presentation, he was suffering from a variety of persistent symptoms: chronic fatigue (level three), dyspnea (level three), anosmia (level five), loss of taste (level five), muscle weakness (level four), difficulty sleeping (level one) and headaches (level two). The nicotine-naïve patient agreed to the off-label use of nicotine patches in the manner previously described.

Unfortunately, the patient did not administer the recommended dose of 7.5 mg/24 h but mistakenly doubled it (15 mg/24 h), which led to intolerable vomiting (level five) and diarrhea (level five) within seven hours; the patient discontinued the therapy after ten hours.

Despite the cessation of nicotine use, chronic fatigue decreased significantly on day two after nicotine use (stage two), continued to decrease on day three (stage one) and was no longer detectable on day four. Similar to the cases described previously, the symptoms of anosmia and loss of taste showed a rather protracted yet continuously declining course; the reduction of these two symptoms occurred simultaneously in this particular case. On the eleventh day after nicotine application, there was a slight reduction in both symptoms (level four), which then dropped to level three on the twelfth and thirteenth days. After a decrease to level two on the fourteenth day, the patient was able to fully perceive all smell and taste qualities on the fifteenth day.

The mild sleep problems (level one) reported by the patient permanently disappeared on the first day following nicotine patch application (level zero). Regarding the feeling of weakness, the patient described a daily reduction by one level, reaching level one on the third day; this lasted for one more day, reaching permanent elimination from day five onwards (level zero). The residual headache reported by the patient (level two) was completely resolved by day two after nicotine administration (level zero). This patient also reported no recurrence of the described symptoms after an interval of six months.

### Case 4

A 52-year-old male patient presented to our outpatient clinic on April 1^st^, 2022, stating that he had been suffering from persistent complaints including chronic fatigue (level two), shortness of breath (level two), difficulty concentrating (level one), difficulty sleeping (level three), mood swings (level two), chest tightness (level two) and palpitations (level two) since a PCR-positive SARS-CoV-2 infection on March 3^rd^, 2022.

After excluding persistent acute SARS-CoV-2 infection via a negative PCR test, we informed the patient of the apparent presence of post-COVID-19 syndrome. The nicotine-naïve and otherwise healthy patient agreed to a therapy trial using a nicotine patch (7.5 mg/24 h). Without consultation and contrary to our recommendations, the patient increased the nicotine dose to 15 mg/24 h on the third day of therapy; he then stopped the application completely on the fourth day after nearly complete symptom remission. He stated that he had not experienced any side effects of the nicotine application, which is why he doubted the efficacy and therefore applied two nicotine patches at 7.5 mg/24 h each starting on day three.

Chronic fatigue increased slightly on the second day of nicotine application (level three) and then decreased significantly on day five (level one). On the sixth day, fatigue was no longer reported. The complaints of breathlessness dropped on day five (level one) and were no longer reported from day seven onwards (level zero). The patient reported that the concentration difficulties had ceased on the first day of nicotine use (level zero). Difficulty sleeping and mood swings persisted until the fourth day (level two) and were no longer detectable from the fifth day onwards (level zero).

The perceived chest tightness (level two) dropped on day five (level one) and was no longer detectable on the following day (level zero). The intermittent palpitations (level one), which were perceived as mild, had not occurred for two days once nicotine usage had begun. On day three of therapy, the patient once again noticed episodes of palpitations (level two), which were recorded at this level for two days. On day three of nicotine administration, this discomfort (level one) dropped and was gone completely on day four. We interpreted this recurrence of palpitations as a classic side effect of nicotine; it stopped spontaneously and did not require further treatment. In an interview three months after the intervention, the patient confirmed that he had not noticed any recurrence of the symptoms that had brought about his initial consultation.

## Discussion

Each of the four presented cases showed significant alleviation of their persistent symptoms; improvement was reached either immediately following nicotine patch application or in rapid succession after treatment began. There were clear differences in the patterns and the time spans for symptom relief among the four cases. It is also worth noting that the course of symptom improvement in each of the presented cases was independent of their drastically different lengths and progression prior to nicotine therapy.

In each case, signs of exhaustion such as fatigue, weakness, breathlessness and exercise intolerance improved rapidly and across the board following nicotine exposure (at the very latest by day six). In cases with impairment or loss of the senses of taste and smell, improvement was observed over a longer period, with complete restoration of these senses over anywhere from thirteen to sixteen days.

The perceived tightness in the chest, as well as palpitations, were described as clear (level two) and ended on the second day after their occurrence (day three after the start of nicotine administration). Regarding the chest tightness described in case two, the patient stated that she had not felt any reduction in performance; this made a coronary and/or vascular nature of the problem seem unrealistic from the author’s point of view. The patient documented complete recovery from this side effect on the twenty-second day after having started nicotine therapy.

The amount of virally blocked AChR can vary greatly among individuals, which certainly influences the course of symptom reduction; this may require individualized nicotine doses and application intervals to suit the individual patients. All cases described were observed in non-smokers. We observed severe side effects only in the patient who had mistakenly applied double the recommended dosage of nicotine. Severe nausea in connection with sweating and repeated vomiting are classic side effects of nicotine and are why this patient discontinued the therapy. With continuous nicotine abstinence after this dosage misstep, all COVID-19-related symptoms previously documented by the patient decreased until restitutio ad integrum was achieved on the fifteenth day after nicotine application. From the author's point of view, this development supports the underlying hypothesis, as SARS-CoV-2 displacement from nAChR binding locations should follow a certain dose–response relationship.

In the case of the patient who independently doubled the recommended dose starting on day three (case four), we suspect that the administration of the recommended dose may have led to a habituation reaction that helped lessen potential side effects from the higher dosage.

The release of the SARS-CoV-2 virus from nAChR receptors can lead to short-term viremia with signs of acute SARS-CoV-2 infection when starting nicotine therapy; however, this viral load should be neutralized within a short period of time by the humoral component of the immune system due to SARS-CoV-2 antibodies formed during the acute phase of infection (Fig. 2C,D).

Transcutaneous administration of nicotine ensures constant serum levels without relevant peak levels. Thus, we did not see any development of nicotine dependence in the context of nicotine patch therapy. From the author's point of view, this is not to be expected.

The overwhelming similarity between the large number of post-COVD-19 syndrome symptoms and the well-known central and peripheral symptoms of the central anticholinergic syndrome (Heck and Fresenius 2001) encourages the author to believe that long-haul COVID must be a profound cholinergic signal transmission disorder. Caused by a significantly higher affinity of SARS-CoV-2 to the nAChR compared to the natural ligand ACh (Oliveira et al. 2021), its displacement from AChRs with subsequent blockade of the intrinsic activity of ACh on the nAChR help explain the myriad of typically reported symptoms.

The cases presented describe patients who had no co-morbidities alongside their post-COVID-19 syndrome. Therefore, the non-critical use of nicotine patches in patients with relevant cardiovascular or respiratory diseases, or those with existing medication regimens, is not advisable. For patients such as these, it is safer to apply nicotine under inpatient conditions.

The presentation of four individual case descriptions does not allow for general conclusions to be made about the therapeutic effect of transcutaneous nicotine administration in post-COVD-19 syndrome; this would require double-blinded, randomized studies. Due to the minimal therapeutic intervention, studies such as these have the potential to be carried out rather easily.

Due to the lack of blinding, the author believes that the psychosomatic component, which other authors suspect to be a central component of long-haul COVID (Stengel et al. 2021), cannot be safely ruled out as part of the therapeutic effect. However, no symptom-related relapses were observed in a follow-up telephone consultation with the patients three to six months post-intervention.

The studies conducted by Changeux et al. (2020) and Alexandris et al. (2021) show the high structural and functional affinity of the corresponding SARS-CoV-2 SGP sections to the nAChR, without making a quantitative comparison to the dissociation constants (K_i_) of ACh and nicotine (Changeux et al. 2020; Alexandris et al. 2021). Therefore, the displacement of ACh from nAChR binding locations by SARS-CoV-2, along with the removal of this blockade via nicotine, remains speculative. The assumption of this constellation is based solely on the well-known, much higher affinity of nicotine for the nAChR when compared to ACh.

Investigations that follow such a quantitative approach would be necessary to objectify the hypothesis put forward by the author. Considering the substantial burden on health care systems and the expected high incidence of post-COVID-19 syndrome, coupled with the currently extraordinarily long courses of therapy and their unpredictable results, this treatment approach is worth investigating further. The low therapeutic effort of a nicotine patch and the easily controllable side effects of a well-known substance seem to justify carrying out larger, double-blinded and randomized investigations based on the described hypothesis. Considering that the previous attempts at explaining the etiopathogenesis of long-haul COVID, and developing appropriate therapeutic efforts, have been speculative, it is worth looking into this hypothesis more closely. Nicotinic AChRs and specifically the a7nAChR play a major role in the vagus nerve regulation of inflammation through the efferent arm of the inflammatory reflex (Wang et al. 2003; Pavlov 2019; Tracey 2002) and there is a growing interest in exploring electrical vagus nerve stimulation in neuromodulation strategies to control inflammation and treat several chronic diseases under the umbrella of the growing field of Bioelectronic Medicine (Pavlov and Tracey 2022; Pavlov et al. 2020). There is also an interest in using non-invasive VNS in the treatment of COVID and post-COVID syndromes (Pavlov and Tracey 2022; Pavlov 2021).

## Conclusions

Post-COVID-19 syndrome is well explained in its pathogenesis and clinical manifestation, with cholinergic neuromodulation disorder due to partial or complete blockage of nicotinic acetylcholine receptors by the SARS-CoV-2 virus playing a potentially important role. In all four of the cases we studied, transcutaneous use of nicotine led to a near immediate improvement in symptoms and rapid restitutio ad integrum. The course of symptom improvement was as distinct as the clinical presentation of post-COVID-19 syndrome in each patient. The ease of implementation and the good controllability of the minor side effects make randomized, double-blinded studies to investigate this treatment option more closely seem feasible. Based on the results of this case study, this treatment option—using nicotine patches to combat long-haul COVID—seems far superior to the time-consuming, often underwhelming or disappointing, costly and complex rehabilitation measures currently available to these patients.

## Supplementary Information

**Additional file 1.**

**Additional file 2.**

**Additional file 3.**

**Additional file 4.**

## Data Availability

Not applicable.
