# Supplementary figures and images for: Is the post-COVID-19 syndrome a severe impairment of acetylcholine-orchestrated neuromodulation that responds to nicotine administration?
Source: Bioelectron Med. 2023 Jan 18;9:2. doi: 10.1186/s42234-023-00104-7 (PMC9845100; doi:10.1186/s42234-023-00104-7)

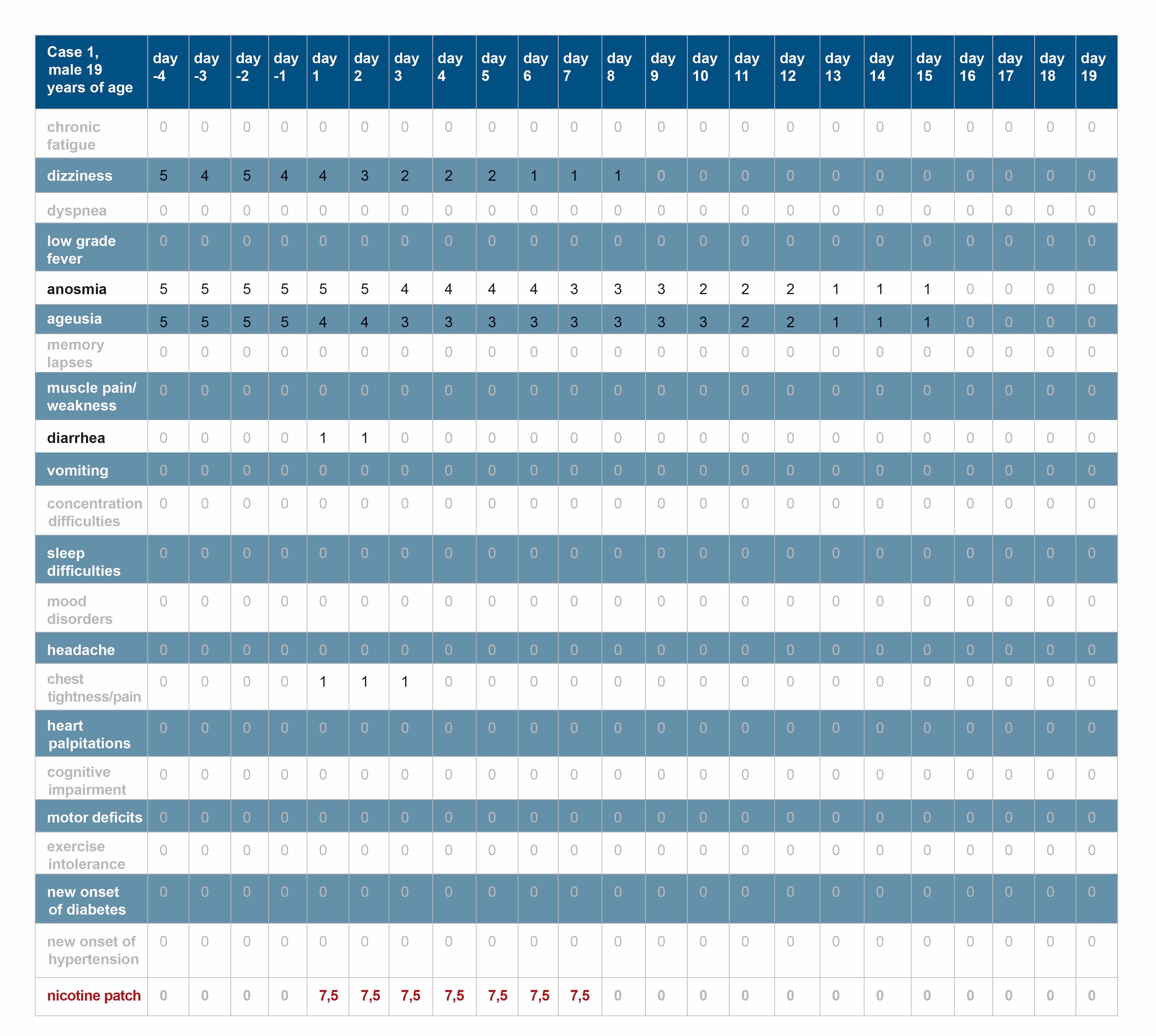

Supplement: Supplementary file 1 — Additional file 1. [file 42234_2023_104_MOESM1_ESM.jpg]

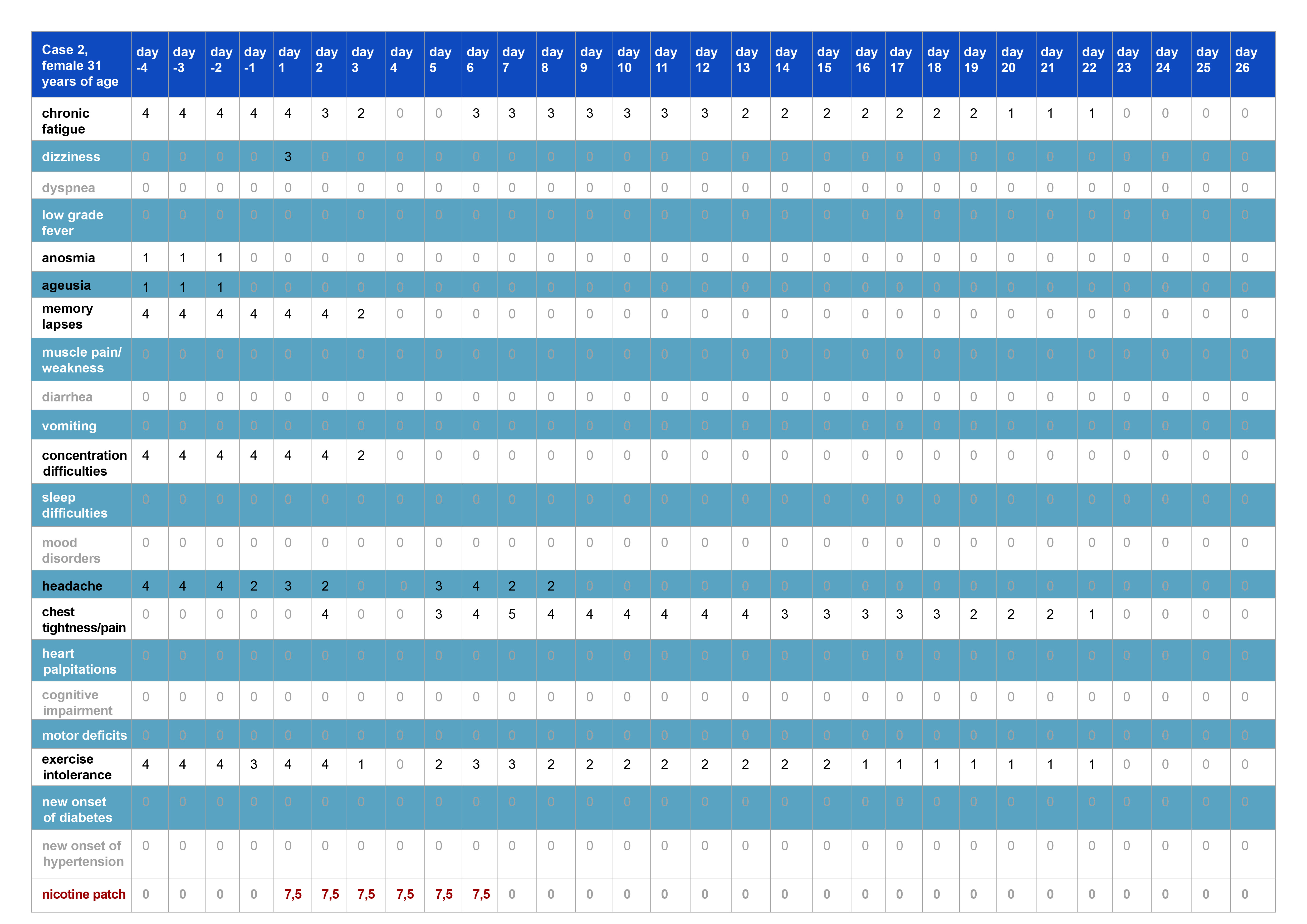

Supplement: Supplementary file 2 — Additional file 2. [file 42234_2023_104_MOESM2_ESM.jpg]

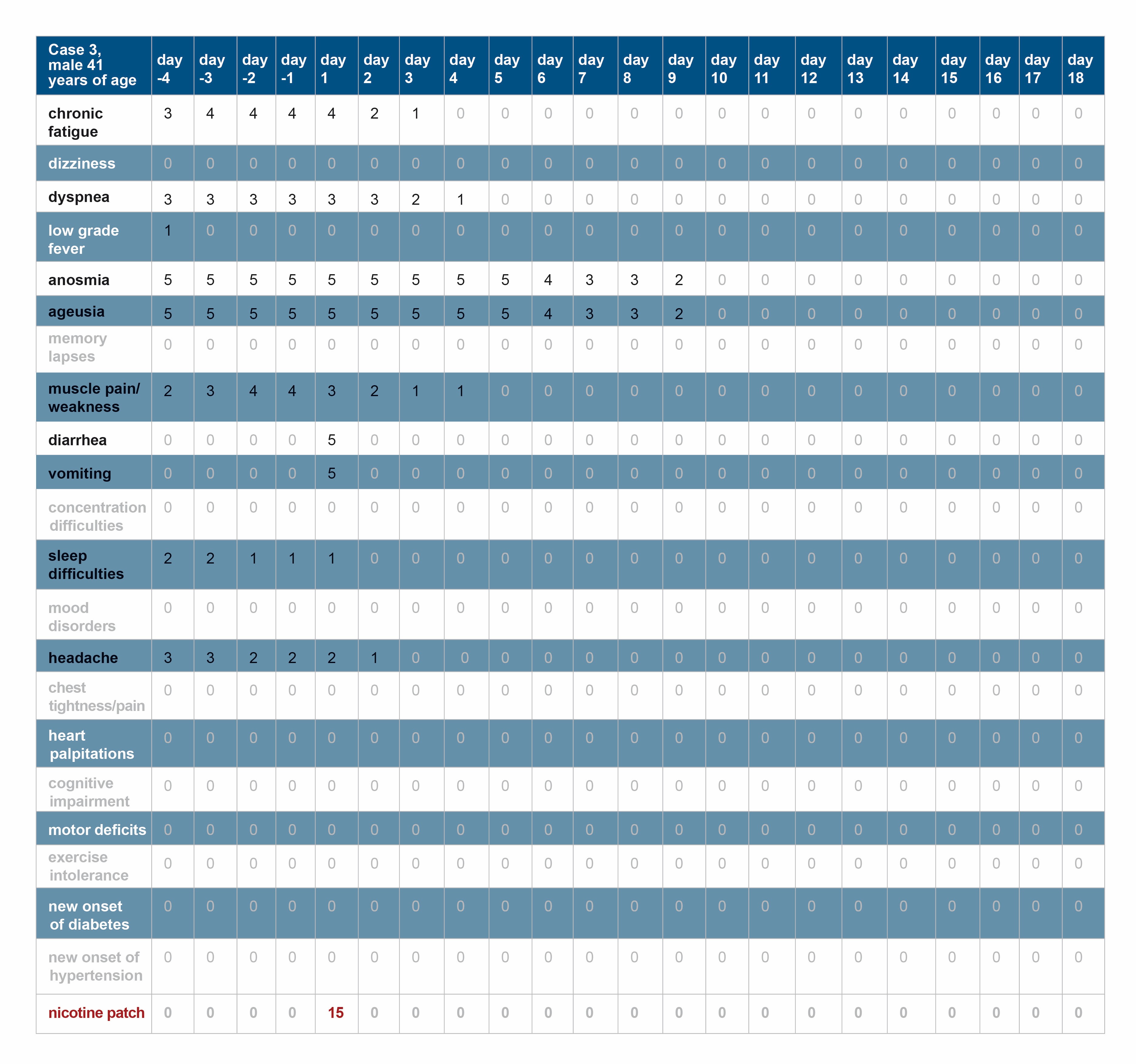

Supplement: Supplementary file 3 — Additional file 3. [file 42234_2023_104_MOESM3_ESM.jpg]

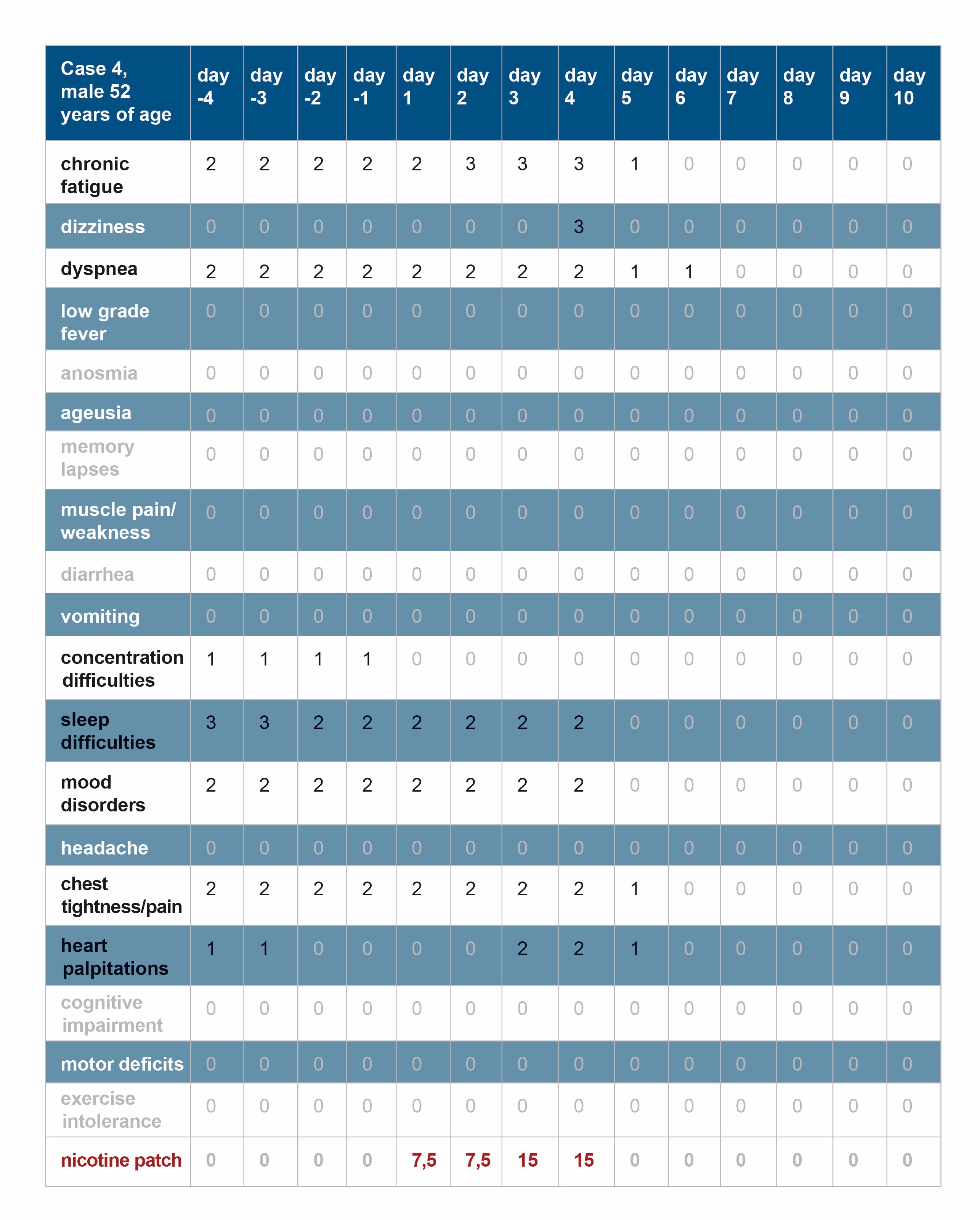

Supplement: Supplementary file 4 — Additional file 4. [file 42234_2023_104_MOESM4_ESM.jpg]
